# Lost in Translation: A Disconnect Between the Science and Medicare Coverage Criteria for Continuous Subcutaneous Insulin Infusion

**DOI:** 10.1089/dia.2021.0196

**Published:** 2021-09-30

**Authors:** Grazia Aleppo, Christopher G. Parkin, Anders L. Carlson, Rodolfo J. Galindo, Davida F. Kruger, Carol J. Levy, Guillermo E. Umpierrez, Gregory P. Forlenza, Janet B. McGill

**Affiliations:** ^1^ Division of Endocrinology, Metabolism and Molecular Medicine, Feinberg School of Medicine, Northwestern University, Chicago, Illinois, USA.; ^2^ Clinical Research, CGParkin Communications, Inc., Henderson, Nevada, USA.; ^3^ International Diabetes Center, Endocrinologist, Regions Hospital & HealthPartners Clinics, Minneapolis, Minnesota, USA.; ^4^ Diabetes Education Programs, HealthPartners and Stillwater Medical Group, Minneapolis, Minnesota, USA.; ^5^ University of Minnesota Medical School, Minneapolis, Minnesota, USA.; ^6^ Emory University School of Medicine, Atlanta, Georgia, USA.; ^7^ Center for Diabetes Metabolism Research Emory University Hospital Midtown, Atlanta, Georgia, USA.; ^8^ Hospital Diabetes Taskforce, Emory Healthcare System, Atlanta, Georgia, USA.; ^9^ Division of Endocrinology, Diabetes, Bone & Mineral, Henry Ford Health System, Detroit, Michigan, USA.; ^10^ Division of Endocrinology, Diabetes, and Metabolism, Icahn School of Medicine at Mount Sinai, New York City, New York, USA.; ^11^ Mount Sinai Diabetes Center and T1D Clinical Research, Icahn School of Medicine at Mount Sinai, New York City, New York, USA.; ^12^ Division of Endocrinology, Metabolism Emory University School of Medicine, Atlanta, Georgia, USA.; ^13^ Diabetes and Endocrinology, Grady Memorial Hospital, Atlanta, Georgia, USA.; ^14^ Barbara Davis Center, Division of Pediatric Endocrinology, Department of Pediatrics, University of Colorado Denver, Denver, Colorado, USA.; ^15^ Division of Endocrinology, Metabolism and Lipid Research, Washington University in St. Louis, School of Medicine, St. Louis, Missouri, USA.

**Keywords:** CSII, AID, HCL, LGS, PLGS, Centers for Medicare & Medicaid Services, Insurance coverage, Type 1 diabetes, Type 2 diabetes

## Abstract

Numerous studies have demonstrated the clinical value and safety of insulin pump therapy in type 1 diabetes and type 2 diabetes populations. However, the eligibility criteria for insulin pump coverage required by the Centers for Medicare & Medicaid Services (CMS) discount conclusive evidence that supports insulin pump use in diabetes populations that are currently deemed ineligible. This article discusses the limitations and inconsistencies of the insulin pump eligibility criteria relative to current scientific evidence and proposes workable solutions to address this issue and improve the safety and care of all individuals with diabetes.

## Introduction

As the prevalence of diabetes continues to increase, managing the costs associated with treating the disease and its complications is problematic for government and commercial payers, worldwide. In the United States, the total estimated cost of diagnosed diabetes exceeded $327 billion in 2017,^1^ with the cost of undiagnosed diabetes estimated at almost $32 billion.^2^ Approximately $180 billion (55%) of these costs for diagnosed diabetes are related to treating the complications of diabetes that arise from suboptimal glycemic control. These costs include inpatient hospitalizations ($69.6 billion), emergency department/ambulance services ($8.3 billion), hospital outpatient care ($12.0), and lost productivity ($90.0 billion),^1^ whereas the cost of medical equipment, including glucose monitoring devices and insulin pumps, comprise only $1.3 billion (0.4%).

The increasing prevalence of diabetes is worrisome in the Medicare population. Approximately 26.8% of U.S. adults at age ≥65 years have diabetes.^3^ Within the Medicare population, the prevalence of diabetes was 31.6% according to the latest estimates (2015).^4^ Within this population, the average per capita cost of diabetes is two times greater than individuals at age <65 years, $13,239 versus $6675, respectively.^1^

Treatment of older insulin-treated diabetes patients is particularly challenging because of their comorbidities, including cardiovascular and kidney disease, cognitive decline, and orthopedic problems causing impaired mobility. Older patients with diabetes of long duration are more likely to need insulin therapy and are at increased risk for hyperglycemia, severe hypoglycemia, and glucose variability and have a higher prevalence of impaired hypoglycemia awareness.^5–10^ The increased risk of severe hypoglycemia among patients ≥65 years occurs with both intensive insulin regimens and with less intensive insulin regimens or oral antidiabetic medications.^11–13^

As reported by Lipska et al. in a retrospective analysis of Medicare beneficiaries, data from 1999 to 2011 showed an 11.7% increase in inpatient admission rates for severe hypoglycemia (from 94 to 105 admissions per 100,000 person-years).^14^ In 2011 the average cost per hypoglycemia hospitalization was $10,139,^15^ which equates to $13,265 when inflated to US$ 2021.^16^

The logical approach to improving outcomes and reducing these costs would be to encourage greater use of tools that have been proven effective in addressing dysglycemia, including elevated HbA1c levels and excessive glycemic variability leading to hypoglycemia. All are significant risk factors for developing acute^17–19^ and chronic^19–23^ complications of diabetes. Among these tools is insulin pump therapy.

Numerous studies have demonstrated the efficacy of insulin pump use, with and without continuous glucose monitoring (CGM) in individuals with type 1 diabetes (T1D).^24–32^ However, a growing body of evidence shows similar benefits in individuals with type 2 diabetes (T2D) who are treated with multiple daily insulin injections (MDI).^33–44^

Although the majority of T2D studies have shown only short-term benefits of insulin pump therapy, a 5-year follow-up study of insulin pump use in T2D adults showed persistent glycemic benefits in this population.^33^ Another example is the prospective, crossover trial by Chlup et al., which showed that T2D adults previously treated with MDI therapy who initiated insulin pump therapy experienced significant HbA1c reductions (−0.9%), whereas those who remained on MDI therapy showed no improvement at 6 months.^43^ At 12 months, earlier MDI users who crossed over to insulin pump showed significant HbA1c reductions (−0.5%), whereas those who continued insulin pump therapy achieved an additional HbA1c reduction (−0.7%).

The safety and efficacy of insulin pump therapy in patients with T2D were also demonstrated in the Opt2mise program, in which participants who failed to achieve adequate glycemic control on MDI therapy achieved significant, sustained improvements in glycemic outcomes when switched to insulin pump therapy.^35^ In the recent VIVID study that assessed glycemic variability and time in optimal glucose range in a subset of T2D adults who required high doses of insulin, Blevins et al. reported that participants using continuous subcutaneous insulin infusion (CSII) experienced significant reductions in glycemic variability compared with those treated with MDI.^45^

The efficacy of insulin pump therapy in reducing hypoglycemia risk in older patients was demonstrated in a study by Yeoh et al. that assessed changes in HbA1c and hypoglycemia in 34 patients, >65 years of age.^46^ Significant reductions in HbA1c were observed at 1 year and sustained for up to 9 years. Of importance, in the 28 patients with documented status of hypoglycemia awareness, the proportion of patients reporting impaired awareness decreased from 50% to 40.6% during the first year of insulin pump use. Among the 22 patients who initiated insulin pump therapy because of disabling hypoglycemia, the proportion of patients reporting ≥1 episode at baseline decreased from 58.3% to 16.7%.

We also have a new generation of insulin-delivery technologies that can detect and or predict dangerous glycemic events and then automatically suspend or increase insulin infusion. These new devices both improve overall glycemic control and reduce hypoglycemia, which numerous studies have shown to be common occurrences and a significant risk factor for hospitalizations and mortality among older adults with diabetes.^5–10^ Moreover, these systems have been shown to increase the time spent in each user's target glucose range, reducing time spent in hyperglycemic and in hypoglycemic ranges, which are risks not detected through HbA1c testing. Frequent and/or excessive glycemic variability^47^ poses significant risks for acute and chronic diabetes complications.^48^

As demonstrated in the 2018 Tandem Basal-IQ study involving 103 T1D participants (age 6–72 years), use of the Tandem Diabetes Care Basal-IQ predictive low-glucose suspend (PLGS) system (Tandem Diabetes Care, Inc., San Diego, CA) integrated with a Dexcom G5 sensor (Dexcom, Inc., San Diego, CA) and a PLGS algorithm resulted in significantly reduced hypoglycemia without rebound hyperglycemia, demonstrating that the system can improve glycemic control in youth and adults.^49^ More recently, real-world data from 9451 Tandem Control-IQ users showed increased time in glucose range with reduced time above range over 12 months.^50^ Most of these individuals were using the Basal-IQ system before the Control-IQ.

The MiniMed 780G US Pivotal Trial was a single-arm study that compared the sensor-augmented Medtronic MiniMed 780G Advanced Hybrid Closed Loop (AHCL) (Medtronic, Inc., Northridge, CA) with sensor-augmented pump therapy or MiniMed 670G in a cohort of 157 T1D adolescents and adults.^51^ At 3 months, use of the AHCL was associated with reduced hypoglycemia and improved HbA1c. Similar glycemic benefits were reported with use of the OmniPod 5 (Insulet Corp., San Diego, CA)^52^ and the Loop open source hybrid closed loop system (LoopDocs).^53^ Note that insulin-requiring patients with T2D on MDI therapy currently qualify for the use of CGM, but most Medicare beneficiaries do not qualify for insulin pump use.

It is, therefore, unfortunate that many Medicare beneficiaries with insulin-treated diabetes who would benefit from insulin pump use are denied access to this technology because of onerous, medically unfounded eligibility criteria. This article discusses the limitations and inconsistencies of the Medicare coverage requirements relative to current scientific evidence and proposes workable solutions to address this issue and improve the safety and care of all individuals with diabetes.

## Current Eligibility Criteria Are Based on Outdated or No Scientific Evidence

When making its determination for coverage of insulin pump therapy for Medicare beneficiaries^54^ (Table 1), the Centers for Medicare & Medicaid Services (CMS) performed a rigorous review of the literature. As detailed in their document, Decision Memo for Insulin Pump: C-Peptide Levels as a Criterion for Use (CAG-00092R),^55^ the agency considered the available relevant studies and professional society consensus statements to determine whether the evidence was of sufficient quality to support insulin pump coverage. However, because the determination was made in 2004, the most recent and relevant studies were, by default, excluded from their assessment.^24–43,56–58^

**Table 1.** Current Medicare Eligibility Criteria for Insulin Pump Coverage^54^

Insulin pumps are covered as medically reasonable and necessary in the home setting for individuals with diabetes who (1) either meet the specified fasting C-peptide testing requirement, or, are beta cell autoantibody positive; and, (2) satisfy the remaining criteria for insulin pump therapy as described hereunder:^54^
• Has completed a comprehensive diabetes education program
• Documentation of treatment with multiple daily injections of insulin (≥3 injections daily) for at least 6 months before initiation of the insulin pump
• Documentation of blood glucose monitoring frequency (≥4 tests daily) during the 2 months before initiation of the insulin pump. This also applies to beneficiaries who have been on insulin pump therapy before Medicare enrollment
• Meets one or more of the following requirements:
○ >7.0% HbA1c
○ Documented history of recurring hypoglycemia
○ Documentation of wide fluctuations in blood glucose before mealtime
○ Documentation of dawn phenomenon with fasting blood glucose frequently >200 mg/dL
○ Documentation of history of severe glycemic excursions
• Continued coverage requirements:
○ The beneficiary is seen and evaluated by the treating physician at least every 3 months
○ The pump must be ordered by and follow-up care of the patient must be managed by a physician who manages multiple patients using insulin pumps and who works closely with a team including nurses, diabetes educators, and dietitians who are knowledgeable in the use of insulin pumps

As a result, the clinical efficacy of today's advanced insulin-delivery technologies integrated with continuous glucose sensor technology were not considered. Moreover, these outdated criteria do not reflect important current evidence that is unsupportive or clearly refutes many of the original criteria, such as documentation of insulinopenia, frequent blood glucose monitoring (BGM), and quarterly in-person health care provider evaluation. These onerous and unnecessary requirements severely impede beneficiary access to the proven benefits of insulin pump therapy. The following is a point-by-point assessment of the validity of the current eligibility criteria.

### Proof of insulinopenia is irrelevant to success with insulin pump therapy

According to the Medicare Decision Memo for Insulin Pump use:^55^

*CMS has determined that the evidence is adequate to conclude that continuous subcutaneous insulin infusion (CSII) is reasonable and necessary for treatment of diabetic patients: (1) who either meet the updated fasting C-peptide testing requirement or are beta cell autoantibody positive; and (2) who satisfy the remaining criteria for insulin pump therapy detailed in the Medicare National Coverage Determinations Manual.*

This requirement was drafted almost two decades ago and our understanding of the relevance of C-peptide assessment has evolved to the point where this requirement is no longer consistent with the science. Moreover, these tests are costly, add no value to clinical decision-making, and impose additional burdens on patients and their health care providers.

#### Limitations of C-peptide and islet antibody assessments

Although a fasting C-peptide test is a useful indicator of beta cell function and has been shown to correlate with diabetes type, duration of T2D, and age of diagnosis,^59^ the accuracy of this measurement can be impacted by a number of factors. The presence of a large numbers of anti-insulin antibodies that bind both proinsulin and C-peptide can yield a falsely high C-peptide reading.^59^ Moreover, studies published after the Medicare requirement have shown the persistence of C-peptide levels in individuals with T1D.^60–63^

The limitations of C-peptide testing in T2D are even more profound. As demonstrated in a recent study by Khan et al. a higher C-peptide level is a strong marker for insulin resistance^64^ and is consistently associated with cardiovascular and all-cause mortality in people without diabetes.^65–67^ Therefore, one would expect to see higher C-peptide levels in individuals with T2D, given the high prevalence of insulin resistance and obesity in these individuals.^68^ Conversely, as recently reported by Christensen et al., low C-peptide levels are associated with increased glycemic variability and hypoglycemia risk.^69^

To our knowledge, no study has shown a correlation of islet autoantibody levels and glycemic control. In fact, a recent study by Gumus et al. showed that islet antibody status was not associated with differences in insulin, C-peptide levels, or HbA1c levels.^70^ Moreover, use of islet antibody testing limits insulin pump use to patients with autoimmune diabetes, which excludes most insulin-requiring patients with T2D, and certainly excludes those with insulin deficiency from other conditions such as pancreatic disease or resection and insulin-requiring patients with other types of diabetes, including gestational diabetes or T2D in pregnancy, where tight glucose control is mandatory.

#### No relationship between C-peptide/islet antibody measures and outcomes with insulin pump therapy

Although testing for C-peptide and islet or beta cell antibody levels may be valuable for diagnostic purposes, these measurements are irrelevant to whether an individual would benefit from insulin pump therapy. Current clinical practice guidelines recommend insulin use based on glycemic status and not on insulin-producing capacity or antibody status.^71^ Therefore, it does a disservice to require patients to wait until they reach insulinopenia before offering therapy that could improve glycemia and reduce complications. As demonstrated in numerous studies, the glycemic benefits of insulin pump therapy are not dependent on detection of C-peptide concentrations or detection of autoantibodies.^34–36,56,72–74^

In a retrospective study that assessed a propensity score-matched cohort of insulin pump and MDI users, Gill et al. investigated the differences in health care resource utilization and costs between individuals with T1D and T2D treated with insulin pump or MDI therapy who met or did not meet the Medicare C-peptide/beta cell antibody eligibility criteria.^56^ For these comparisons, investigators structured the study as three analyses. Results from the first analysis showed significantly slower growth in hospital admissions and average annual medical costs among insulin pump users who did not meet the C-peptide/beta cell antibody test criteria compared with those who did, although changes in total medical costs were similar. Although results from the second analysis showed slower growth in inpatient costs at 1 year, similar changes in total medical costs were observed.

In both analyses, insulin pump adopters who did not meet Medicare test criteria showed numerically slower growth in inpatient, outpatient, emergency department costs, and hospital admissions compared with analogous MDI groups. In the third analysis, slower growth in hospital admissions was observed in the insulin pump group compared with MDI therapy, and per-patient annual savings ranged from $201 for emergency department costs to $5133 for total medical costs.

In the OpT2mise trial, a randomized, controlled, multicenter study of 331 T2D adults with suboptimal glycemic control (>9.0% HbA1c), participants were randomized to insulin pump or MDI therapy and were followed for 6 months.^35,72^ Among the study participants, 18% were positive for anti-glutamic acid decarboxylase antibody (anti-GAD Ab) levels and 24% had undetectable C-peptide levels. Of importance, baseline HbA1c values were not correlated with either anti-GAD Ab or C-peptide concentrations. At 6 months, the mean HbA1c in the insulin pump group had decreased 1.1% versus 0.4% in the MDI group, with a between-group difference of −0.7% (*P* < 0.0001).

However, there was no association between HbA1c reductions and C-peptide concentration in both the insulin pump and MDI groups. Regardless of the insulin delivery method used, the mean HbA1c value decreased equally in each anti-GAD Ab category and in each C-peptide quartile. However, the magnitude of HbA1c reduction was always greater in individuals using an insulin pump compared with MDI therapy.

Vigersky et al. showed similar improvements in HbA1c, total daily insulin dose, and treatment satisfaction among T2D patients ≥65 years using insulin pump therapy compared with MDI irrespective of baseline C-peptide level.^73^

In a study of 331 T2D adults who were randomized to insulin pump or MDI therapy, participants were categorized into two groups according to baseline C-peptide level: Group A (≤183 pmol/L) and Group B (>183 pmol/L). At 6 months, the MDI group crossed over to insulin pump therapy. Between- and within-group comparisons were assessed at 6 and 12 months in the entire cohort and separately for those patients ≥65 years of age. Significant HbA1c reductions were observed at both 6 and 12 months among insulin pump users in both C-peptide groups along with reductions in total daily insulin dosages and improvements in treatment satisfaction. Similar findings were reported in the subgroup of older participants.

The evidence clearly does not support the continued use by Medicare of either the C-peptide or beta cell antibody assessment. Their use precludes many beneficiaries from adopting this important technology. Worse, many individuals who have been users of insulin pump therapy for years are denied coverage for this therapy when failing to meet the C-peptide or beta cell antibody standards when aging into Medicare, significantly disrupting their ability to successfully manage diabetes.

### No evidence to support requirement for history of MDI therapy

Given that the majority of individuals with T1D are currently treated with either MDI or insulin pump therapy, this requirement specifically targets those with T2D. In our review of the T2D studies that showed glycemic benefits with insulin pump use, we found two early studies that demonstrated improved outcomes in individuals with T2D who were treated insulin pump therapy after earlier treatment with less-intensive insulin therapy or oral medications.^36,38^ Although recent studies, such as the OpT2mise trial,^34,35^ listed prior MDI therapy in their inclusion criteria, this requirement was for study purposes only.

Although we agree that everyone who initiates insulin pump therapy should be well-trained in intensive insulin therapy, requiring use of MDI for 6 months (an arbitrary timeframe, at best) negatively impacts beneficiaries who develop the requisite skills in a shorter period of time. Again, there is no scientific basis for the current requirement.

### No evidence to support requirement for frequent BGM

Unlike recent CGM studies that definitively showed no association between earlier BGM frequency and glycemic outcomes,^75–80^ we were only able to identify one insulin pump study without CGM use that even reported BGM frequency (mean 2.5 times/day) as a baseline characteristic.^34^ We then reviewed the clinical trials listed as references in the Decision Memo for Insulin Pump: C-Peptide Levels as a Criterion for Use (CAG-00092R).^55^ None of the studies cited in the Memo listed frequency of BGM in their inclusion criteria.^81–84^

Even earlier studies, including the Diabetes Control and Complications Trial^85^ and Lecavalier et al.,^86^ indicated that frequent BGM was not a requirement for inclusion. Therefore, it must be assumed that the current requirement for ≥4 times daily blood glucose testing is not supported in the literature and it is unclear why CMS chose to include unsupported criteria in its coverage policy.

The requirement also fails to consider the potential disruption of therapy for individuals who are newly enrolled in Medicare. According to the criteria, well-controlled individuals who are currently using an insulin pump but transition to Medicare find that only three strips per day are covered, regardless of therapy. Those who do not fulfill the testing requirement before enrollment will lose use of their pump until they satisfy the requirement. This disruption in care will likely result in deterioration of glycemic control. As reported in a study that assessed the impact of the CMS Competitive Bidding program, the subsequent disruption of beneficiary access to their BGM supplies resulted in a significant proportion of beneficiaries who reduced or discontinued BGM use, leading to increased mortality, hospitalizations, and associated costs.^87^

In addition, Medicare's standard coverage for test strips provides coverage of only three test strips per day even for patients needing intensified insulin therapy. To obtain Medicare coverage for four or more test strips per day, beneficiaries, health care providers, and suppliers must provide extensive documentation that demonstrates the medical necessity for additional strips. The documentation requirements are sufficiently complex that they discourage many suppliers from providing more than three test strips per day. Thus, to obtain four or more test strips per day, beneficiaries either pay out-of-pocket for the additional test strips or convince their health care provider to take additional time to document the medical necessity for more frequent testing and negotiate with suppliers to obtain the additional coverage.

### Requiring poor glycemic control punishes motivated beneficiaries

Limiting insulin pump coverage only to beneficiaries with HbA1c >7%, or some other significant dysglycemia event, not only marginalizes those who have been successful in following their self-management regimen but completely ignores current clinical guidelines for individualizing glycemic targets based on each patient's age and health status. In addition, because Medicare requires that beneficiaries have documented problematic glycemia (e.g., recurring hypoglycemia and excessive glycemic variability), beneficiaries who have achieved optimal glycemic control with insulin pump therapy before enrolling in Medicare are denied use of the tool that has enabled them to succeed with their self-management.

Use of CGM, with or without an insulin pump, has abrogated much of the problematic hyper- and hypoglycemia in both MDI and insulin pump users, and this is further improved by the use of insulin pumps with automated insulin delivery. The risks of these life-threatening events return immediately after loss of either of these updated devices. The use of antiquated requirements for life-saving therapies puts patients and health care providers in a bind when patients transition to Medicare from private insurance.

### Quarterly in-person clinical visits are medically unnecessary

The current requirement for quarterly in-person clinic visits is not based on evidence and directly contravenes the current clinical emphasis on individualizing care.^88,89^ Moreover, this requirement has a direct impact on beneficiary health behaviors and clinical outcomes. Results from an assessment of the T1D Exchange registry showed no change in HbA1c levels across different frequency of visits in a population of >600 patients ≥65 years of age.^90^ The requirement also imposes significant and unnecessary burden on Medicare beneficiaries and caregivers related to the visits themselves and to travel and time away from work. In addition, because Medicare will only cover an HbA1c test every 95 days, the discrepancy in visits and laboratory testing adds additional work for patients and health care providers. These inconsistencies in Medicare requirements (testing 4 × per day but only covering three strips per day; visit requirements every 90 days but HbA1c testing every 95 days) causes both patients and health care providers nonproductive time and effort.

In addition, this requirement ignores the utility of telemedicine visits. As reported in a recent systematic review of 19 randomized controlled trials, use of telemedicine was more effective in improving glycemic control and other diabetes-related outcomes than usual care.^91^

The utility of telemedicine visits has also been clearly demonstrated in addressing the challenges created by the COVID-19 pandemic.^92^

### Documentation requirements are burdensome

Changes in documentation requirements are also needed. For example, access to insulin pump therapy is further hindered by the onerous documentation that health care providers are required to submit to obtain coverage for their patients. In a 2017 survey conducted by the American Medical Association (AMA), 92% of the 1000 clinicians surveyed reported that the documentation required to obtain authorization for medications and medical devices both delays patient treatment and negatively affects clinical outcomes.^93^

The current documentation requirements also have direct impact on health behaviors, treatment satisfaction, and medication adherence. As reported by Argento et al. in a 2019 survey of 241 Medicare patients with T1D, 56% reported difficulties in getting their supplies.^94^ The most common complaints reported by respondents were delays in releasing supplies (30%), difficulty in getting paperwork completed by clinicians (24%), and problems scheduling quarterly visits (18%). As a result, 39% of respondents reported changing their pump-related behaviors such as delays in changing infusion sets (66%) and reusing supplies (34%).

These respondents reported significant adverse outcomes, including more erratic glucose levels (48%), more frequent hyperglycemia (17%), increased anxiety or frustration (44%), pain or irritation at infusion set insertion sites (34%), more scarring of sites (28%), and requiring emergency supply shipments (19%) or getting emergency supplies from a local source (15%). When asked to rate their overall experience with Medicare, 28% rated Medicare coverage to be somewhat worse and 31% much worse than earlier insurance. Additional research is needed to fully elucidate the extent to which these issues are impacting patient health and clinician efficiency.

## Medication Adherence Impacts Clinical Outcomes and Health Care Costs

Suboptimal adherence to diabetes medication regimens remains a significant health concern for providers and payers. As demonstrated in recent studies, medication nonadherence leads to poor outcomes,^95,96^ which can increase health care service utilization and overall health care costs.^97–99^ Cutler et al. estimated that the total cost attributable to nonadherence in diabetes in the United States was >$5 billion, with an average per-patient cost ranging from $2741 to $9819.^100^ A 2012 study by Egede et al. found that individuals who are nonadherent to their diabetes medication regimens can have annual inpatient costs 41% higher compared with those who are adherent.^99^

Conversely, optimal medication adherence is associated with improved glycemic control and decreased health care resource utilization.^101,102^ In a 2016 study of 1000 T1D adults (≥65 years), Boye et al. reported a significant association between higher adherence and overall decreased costs, specifically those associated with acute diabetes-related events, and that each 1% increase in adherence was associated with an all-cause cost savings of $65,464 over 3 years.^102^

Ensuring that individuals with T2D are satisfied with their treatment regimen is critical to improving adherence to diabetes medications, particularly in older T2D adults.^103,104^ In a 2018 study that included 135 T2D adults (age 60 years), treatment satisfaction was associated with an increase in adherence to diabetes medications (*β* = 0.34, *P* = 0.02).^104^ In an earlier study that assessed the effect of patient-selected intensive insulin therapy on quality of life in individuals with insulin-treated diabetes, Chantelau et al. reported higher treatment satisfaction among those who were able to choose their intensive insulin regimen (MDI or insulin pump).^105^ Of interest, insulin pump treatment conferred greater protection against hypoglycemia.

Studies have shown that low treatment satisfaction is associated with poor medication adherence.^106,107^ For individuals with insulin-treated diabetes, nonadherence to insulin treatment is often because of the pain and fear associated with injections.^108–110^ As reported by Rubin et al., the prevalence of fear of injections in individuals with insulin-treated diabetes ranges from 10% to 26%.^110^

Because CMS considers diabetes medication adherence to be a critical component of its medication-related quality measures, as evidenced in its Part C and D Star Ratings (D10-Medication Adherence for Diabetes Medications),^111^ it only makes sense that individuals treated with intensive insulin therapy have the option to choose the insulin delivery method that meets their individual needs and preferences. Providing individuals with options for treatment is an essential component of person-centered care.

One remedy for mitigating the issues and obstacles discussed here would be to simplify the eligibility criteria, requiring that only one of two conditions be met (Table 2). The precedence for reliance on clinical judgment was set by CMS in its waiver document issued during the COVID-19 pandemic regarding medical necessity for CGM coverage.^112^ All current criteria, including testing for C-peptide and beta cell antibodies, history of glucose monitoring, frequency of insulin adjustments based on glucose data, prior injection therapy, and in-person clinic visits should be eliminated.

**Table 2.** Proposed Eligibility Criteria for Insulin Pump Coverage

1	Patients already treated with insulin pump therapy before Medicare enrollment
	*OR*
2	Patients with any type of diabetes who require intensive insulin therapy based on the clinical judgment of a licensed health care provider

For documentation purposes, this would only require the health care provider to check a box next to a statement that reads: “The prescribing healthcare provider certifies that the beneficiary is willing and able to consistently test blood glucose using BGM or CGM prior to initiating pump therapy.” This certification would only be required at the time when insulin pump therapy is initiated. It would not be needed for ongoing provision of insulin pump supplies or in cases when a beneficiary was already treated with insulin pump therapy before Medicare enrollment.

The proposed criteria would significantly reduce the administrative burden of obtaining coverage and eliminate current requirements that are unduly restrictive and medically unfounded. In addition, the frequency of visits, either in-person or using telemedicine visits should be reduced from every 3 months to every 6 months, which aligns with the frequency used by most health care providers with their CGM patients. Finally, coverage for test strips should be increased to five strips per day for MDI users and one strip per day for those using CGM.

## Conclusions

Given the importance of health behaviors and medication adherence in daily diabetes self-management, it is incumbent upon health care providers and payers to identify and remove barriers that impede access to proven tools and technologies that enable individuals with diabetes to achieve and maintain optimal glycemic control. As underscored in this article, many of the current Medicare coverage criteria create significant barriers to beneficiaries who could benefit from insulin pump therapy. Significant changes in these criteria are needed.

First, the requirement for laboratory assessments of C-peptide and beta cell autoantibody levels should be eliminated. Well-designed studies that have shown no correlation between success of insulin pump use and C-peptide/beta cell autoantibody levels further support this position.^35,56,72,73^

Second, although frequent glucose testing (BGM or CGM) is necessary for initiating and continuing use of insulin pump therapy, an individual's history of testing frequency before insulin pump initiation should not be a requirement for coverage eligibility. Allowing beneficiaries to choose their insulin-delivery method, in collaboration with their health care provider, is critical to supporting treatment satisfaction, a major driver of treatment adherence and subsequent outcomes.^103–110^ The added safety of insulin pumps with predictive low glucose suspend or automated insulin delivery used in conjunction with CGM should be a strong consideration for insulin pump use by Medicare beneficiaries. Patient choice matters.

Third, the quarterly clinic visit requirement has no scientific basis and should be eliminated. Instead, it should be up to the health care provider's clinical judgment to determine when and how often the beneficiary should be seen. Moreover, patients who are unable to regularly attend their quarterly visits face delays in receiving their supplies on time. The third-party suppliers will not release supplies if there is no chart note and HbA1c every 3 months, which causes further burden to the patient, their health care provider, and the clinic office that needs to provide these notes. Eliminating this requirement would be entirely consistent with the newly announced CMS proposal to lift unnecessary regulations and ease burden on patients and providers.^113^

Changes in documentation requirements are also needed. Access to insulin pump therapy is hindered by the onerous documentation that health care providers are required to submit to obtain coverage for their patients.^93^ The current documentation requirements also have direct impact on health behaviors, treatment satisfaction, and treatment adherence.^94^

According to the most recent CMS data, ∼67% of the insulin-treated Medicare beneficiaries who are currently treated with or will eventually transition to intensive insulin therapy are denied access to insulin pump therapy because of eligibility criteria that are medically unfounded.^87^ From our perspective, insulin pump therapy should be made available to all beneficiaries who would benefit from this technology, are motivated to achieve optimal glycemic control, and have the ability to safely and effectively use their insulin pump for daily diabetes self-management.
